# Comparative effects of remimazolam and propofol on intraoperative redistribution hypothermia in urologic surgery: a retrospective propensity-matched cohort study

**DOI:** 10.7150/ijms.126707

**Published:** 2026-03-30

**Authors:** Ji-Yoon Jung, Woojin Kwon, Tae-Yun Sung

**Affiliations:** Department of Anesthesiology and Pain Medicine, Konyang University Hospital, Konyang University Myunggok Medical Research Institute, Konyang University College of Medicine, 158 Gwangeodong-ro, Seo-gu, Daejeon 35365, Republic of Korea.

**Keywords:** Remimazolam, Propofol, Hypothermia, Urologic surgical procedures, Body temperature, Perioperative care

## Abstract

**Background:**

Redistribution hypothermia, characterized by rapid core-to-peripheral heat transfer after anesthetic induction, is a major cause of perioperative hypothermia and is linked to adverse outcomes. Propofol, widely used for induction, impairs thermoregulatory vasoconstriction. Remimazolam, a novel ultra-short-acting benzodiazepine, provides greater hemodynamic stability, but its thermoregulatory effects remain unclear. This study compared remimazolam and propofol on intraoperative hypothermia in patients undergoing urologic surgery.

**Methods:**

This retrospective observational study included adult patients (≥ 19 years) who underwent elective urologic surgery under general anesthesia between February 2024 and February 2025. Patients receiving remimazolam (0.2 mg/kg) or propofol (1.5-2.5 mg/kg) for induction were classified into respective groups. Propensity score matching (PSM) was performed (1:1 ratio) using demographic and perioperative covariates. The primary outcome was the incidence of redistribution hypothermia, defined as core temperature < 36.0°C within the first hour after induction. Secondary outcomes included severity of hypothermia, maximum temperature decrease, perioperative temperature trends, and predictors of hypothermia.

**Results:**

Among 181 patients analyzed, 71 per group were matched after PSM. Hypothermia incidence was significantly lower with remimazolam than propofol both before (16.5% vs. 50.0%, P < 0.001) and after matching (15.5% vs. 47.9%, P < 0.001). Propofol use was an independent risk factor (adjusted OR: 7.31; 95% CI: 2.81-21.58; P < 0.001). Female sex, higher BMI, and higher baseline temperature were protective factors.

**Conclusion:**

Compared with propofol, remimazolam was associated with lower incidence and severity of redistribution hypothermia in urologic surgery. These findings suggest thermoregulatory benefits of remimazolam, warranting confirmation in prospective trials.

## Introduction

A rapid decrease in core temperature of approximately 0.5-1.5 °C frequently occurs during the first hour after induction of general anesthesia, primarily as a result of internal redistribution of heat from the core to the peripheral tissues. This phenomenon, known as redistribution hypothermia, accounts for most of the early perioperative heat loss and represents a major contributor to inadvertent perioperative hypothermia 1. Perioperative hypothermia has been associated with numerous adverse outcomes, including increased cardiovascular morbidity, altered drug metabolism, excessive bleeding and transfusion requirements, and a higher risk of surgical site infection 2.

Especially in patients undergoing urologic surgery, maintaining normothermia can be particularly challenging. Following the initial redistribution hypothermia (phase I), a slower and nearly linear decline in core temperature (phase II) often occurs, further exacerbated by surgical factors 3. In transurethral procedures and nephrolithotomy, the use of large volumes of irrigation fluids can substantially increase intraoperative heat loss. In addition, surgeries performed in the lithotomy position, such as transurethral procedures, may limit the application of whole-body warming strategies and thereby increase susceptibility to hypothermia 4. Recent studies in urologic surgery have highlighted the continued clinical relevance of perioperative hypothermia and identified several procedure-related risk factors for heat loss 5,6,7. In addition, evidence from transurethral procedures suggests that preventive strategies such as prewarming may attenuate perioperative temperature decline 8,9. Once redistribution hypothermia occurs, it is difficult to treat because it takes a considerable amount of time for the heat to reach the core tissues against the temperature gradient. Thus, prevention of redistribution hypothermia is important 1.

Redistribution hypothermia results from central inhibition of tonic thermoregulatory vasoconstriction in arteriovenous shunts and arterial and venous vasodilation by anesthetics 10,11. Consequently, the choice of induction anesthetic plays a critical role in determining the magnitude of early core temperature redistribution. Propofol is a drug commonly used to induce general anesthesia. Remimazolam is a novel benzodiazepine, an intravenous anesthetic that has been introduced into clinical practice relatively recently. Previous studies comparing propofol and remimazolam have demonstrated differences in their hemodynamic effects, including systemic vascular resistance and cardiac output 12,13. Given that these hemodynamic variables are closely linked to heat redistribution, such differences may plausibly influence the extent of redistribution hypothermia during anesthesia induction 14,15. However, to the best of our knowledge, no study has compared the effects of the two drugs on redistribution hypothermia when used as induction anesthetics.

Therefore, this study aimed to compare early intraoperative core temperature changes and the incidence of redistribution hypothermia during the first hour after induction of general anesthesia between remimazolam and propofol in patients undergoing urologic surgery. A secondary aim was to identify patient- and perioperative factors associated with the development of redistribution hypothermia in this population.

## Materials and Methods

### Study design and patient selection

This retrospective observational study was approved by the Institutional Review Board of Konyang University Hospital, Daejeon, Republic of Korea (Chairperson Prof. JW Son) on 21 April 2025 (KYUH 2025-03-011). This study adhered to the ethical principles outlined in the Declaration of Helsinki. Given its retrospective design, informed consent was waived, and all patient data were anonymized to ensure confidentiality and protect privacy. This study was conducted and reported in accordance with the Strengthening the Reporting of Observational Studies in Epidemiology (STROBE) statement.

We retrospectively reviewed the medical records of patients aged 19 years or older who underwent elective urological surgery under general anesthesia from February 2024 to February 2025. The included procedures comprised transurethral bladder and prostate procedures, endoscopic stone surgeries, and other minimally invasive urologic procedures performed under general anesthesia. Patients who used propofol or remimazolam as an anesthetic for anesthesia induction and whose anesthesia time was 60 to 180 minutes were included in this study. The exclusion criteria were as follows: missing core temperature records during anesthesia, use of propofol or remimazolam for maintenance of anesthesia (i.e., total intravenous anesthesia), requirement of blood transfusion during surgery, pre-induction body temperature below 36.0 °C or above 37.6 °C, obesity (body mass index [BMI] > 35 kg/m²), and the use of a supraglottic airway device.

### Anesthesia protocol

According to our institutional protocols, the ambient temperature in the operating room was maintained at 21-24 °C, and the ambient temperature in the pre-operative holding area and post-anesthesia care unit (PACU) was maintained at 22-25 °C 16. All patients undergoing elective surgery fasted for at least 8 hours and were admitted to the operating room without premedication. All patients in this study received the same anesthetic management, except for the anesthetic induction agent (i.e., propofol or remimazolam) used for anesthesia induction. Therefore, in this study, the subjects were classified into the propofol group or the remimazolam group according to the intravenous anesthetic used to induce anesthesia.

Before induction of anesthesia, routine monitoring including electrocardiography, noninvasive blood pressure measurement, pulse oximetry, Patient State Index (PSI; SedLine®; Masimo Corp., Irvine, CA, USA), and zero-heat-flux thermometer (Bair Hugger™ temperature monitoring system; 3M, St. Paul, MN, USA) was initiated in all patients. Neuromuscular train-of-four (TOF) by acceleromyography was applied to the adductor pollicis muscle of the arm for neuromuacular monitoring.

In the remimazolam group, anesthesia was induced by remimazolam bolus (0.2 mg/kg) and a target-controlled infusion of sufentanil. Remiamzolam was administered slowly over approximately 30 seconds 12, while monitoring the patient's clinical response. When loss of consciousness was deemed insufficient, additional doses of remimazolam (3-5 mg) were administered.

In the propofol group, anesthesia was induced by propofol bolus (1.5-2.5 mg/kg) and a target-controlled infusion of sufentanil. After confirming the patient's loss of consciousness, rocuronium 0.6 mg/kg was administered and endotracheal intubation was performed at TOF count 0. In both groups, the effect-site concentration of sufentanil was set to 0.4 ng/ml before endotracheal intubation, and the effect-site concentration was adjusted to 0.1 ng/ml after endotracheal intubation and maintained throughout the surgery.

After endotracheal intubation, anesthesia was maintained with fixed dose of sufentanil (effect-site concentration 0.1 ng/ml), sevoflurane and 50% air. During surgery, anesthetic depth was continuously monitored using the Patient State Index (PSI; SedLine®; Masimo Corp., Irvine, CA, USA), and sevoflurane concentration was titrated within an age-adjusted minimum alveolar concentration [MAC] range of 0.7-1.5 to maintain a PSI between 25 and 50.

Forced-air warming (set temperature of 38 °C) was applied to the upper body of all patients during anesthesia, and inhaled gas was supplied through a respiratory circuit heated to 39.5 °C and humidified. Intravenous crystalloids administered for perioperative fluid management and irrigation fluids used during transurethral or endoscopic urologic procedures were both administered at room temperature according to institutional routine. After surgery, all patients were extubated and transferred to the PACU. In the PACU, if the patient complained of cold or had a patient temperature below 36 °C, forced-air warming (set temperature 43 °C) was applied.

### Primary and secondary outcomes

Occurrence of redistribution hypothermia was defined as a patient temperature less than 36 °C as measured by a zero-heat-flux thermometer during the first hour after induction of anesthesia. Severity of redistribution hypothermia was classified as mild (35.5-35.9 °C), moderate (35.0-35.4 °C), or severe (≤ 34.9 °C) 16.

The following data were collected from the electronic medical records: age, sex, weight, height, BMI, American Society of Anesthesiologists (ASA) classification, body temperature measured by a zero-heat-flux thermometer (before anesthesia induction, which was defined as the baseline temperature; immediately after induction; every 15 minutes for the first hour; and at the end of surgery), dosage of remimazolam and propofol, patient position during surgery, intraoperative hemodynamic parameters (before induction of anesthesia, every 15 minutes during the first hour after induction, and at the end of surgery), administered vasopressors; intraoperative fluid volume, estimated blood loss, duration of anesthesia, and use of postoperative forced air-warming device. Estimated blood loss was obtained from anesthesia records; however, in minimally invasive urologic procedures, blood loss was often minimal and recorded as small or rounded values rather than measured directly. Total intraoperative intravenous fluid volume was collected from the anesthesia records; however, the exact irrigation fluid volume was not consistently retrievable from the retrospective medical records.

### Statistical analysis

Demographic and intraoperative characteristics were compared between groups before and after propensity score matching (PSM). PSM was performed using the nearest-neighbor method with a 1:1 ratio and a caliper width of 0.2. The following covariates were used for propensity score matching: age, sex, BMI, ASA classification, duration of anesthesia, operation position, estimated blood loss, administered fluid, baseline temperature, ephedrine use, and phenylephrine use. Absolute standardized mean differences (SMDs) were calculated to assess balance between matched groups, with SMDs < 0.1 considered indicative of adequate balance.

Continuous variables are presented as mean ± standard deviation or median (interquartile range), as appropriate, and categorical variables as number (percentage). In the unmatched cohort, between-group comparisons were performed using the Student's t-test or Mann-Whitney U test for continuous variables and the chi-square test or Fisher's exact test for categorical variables. After propensity score matching, paired statistical methods were applied to account for the matched structure of the data. McNemar's test was used for binary outcomes, and paired Wilcoxon signed-rank tests were used for continuous outcomes.

Changes in core temperature over time between groups were analyzed using repeated measures analysis of variance (RM-ANOVA), and the results were visualized using line plots with means and 95% confidence intervals.

To identify factors associated with intraoperative hypothermia, univariate and multivariable logistic regression analyses were performed in the unmatched cohort. Variables with a p-value < 0.2 in univariate analysis were considered candidate predictors, and age and sex were included regardless of univariate significance due to their established relevance in thermoregulation. Final variable selection was performed using backward stepwise elimination.

In the matched cohort, the association between anesthetic group and hypothermia was evaluated using conditional logistic regression stratified by matched pairs as the primary analysis. Because of the limited number of events in the matched cohort, multivariable adjustment after matching was performed as a sensitivity analysis using generalized estimating equations with a logit link and exchangeable correlation structure, clustered by matched pairs.

A post-hoc power analysis was performed using the matched sample size (*n* = 71 in each group), the observed incidence of the primary outcome (47.9% vs. 15.5%), and a two-sided α level of 0.05.

All statistical analyses were conducted using R, version 3.6.3 (R Foundation for Statistical Computing, Vienna, Austria). A two-sided P-value of < 0.05 was considered statistically significant.

## Results

**Figure 1:**
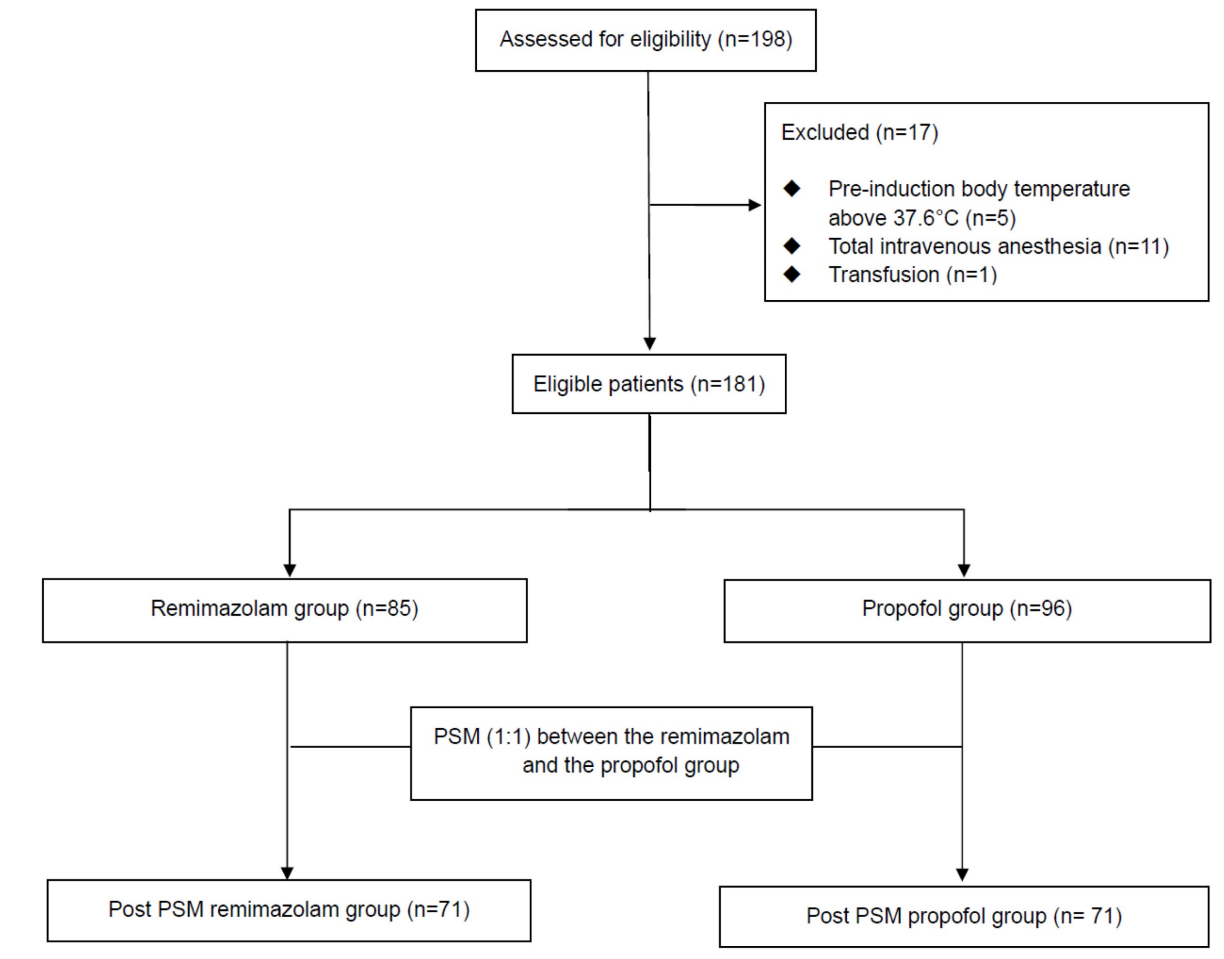
Flow diagram of patient enrollment and propensity score matching.

From February 2024 to February 2025, a total of 198 patients aged 19 years or older who underwent elective urologic surgery under general anesthesia at Konyang University Hospital were assessed. Among them, 17 patients met the exclusion criteria and were excluded, resulting in 181 patients included in the final analysis. After 1:1 propensity score matching, 71 patients were matched in each group (Figure. 1).

Demographic and perioperative data before and after propensity score matching are summarized in Table 1. Before propensity score matching, there were no significant differences in baseline characteristics between the remimazolam (n = 85) and propofol (n = 96) groups. After PSM, 71 patients were matched in each group, and all covariates were adequately balanced, with absolute standardized mean differences < 0.1, including baseline core temperature (SMD = 0.082).

Detailed postoperative outcomes are presented in Table 2. Before PSM, the incidence of intraoperative hypothermia was significantly higher in the propofol group than in the remimazolam group (50.0% vs. 16.5%, P < 0.001). This difference remained consistent after matching (47.9% vs. 15.5%, P < 0.001, McNemar test). With the matched sample size (*n* = 71 in each group) and the observed effect size *h* of 0.719, the calculated statistical power of the study was approximately 99%, indicating that the sample size was sufficient to detect the observed difference between the groups. Additionally, the propofol group exhibited a greater temperature decrease at 60 minutes after induction and required more frequent active warming in the PACU. These findings remained significant after matching based on paired analyses. Also, maximum temperature change, defined as the difference between baseline and the lowest core temperature within 1 hour after induction, was statistically greater in the propofol group than in the remimazolam group, although the absolute difference was modest (P = 0.011). Taken together, remimazolam was associated with both higher absolute core temperatures and a smaller decline from baseline during the early intraoperative period.

**Figure 2:**
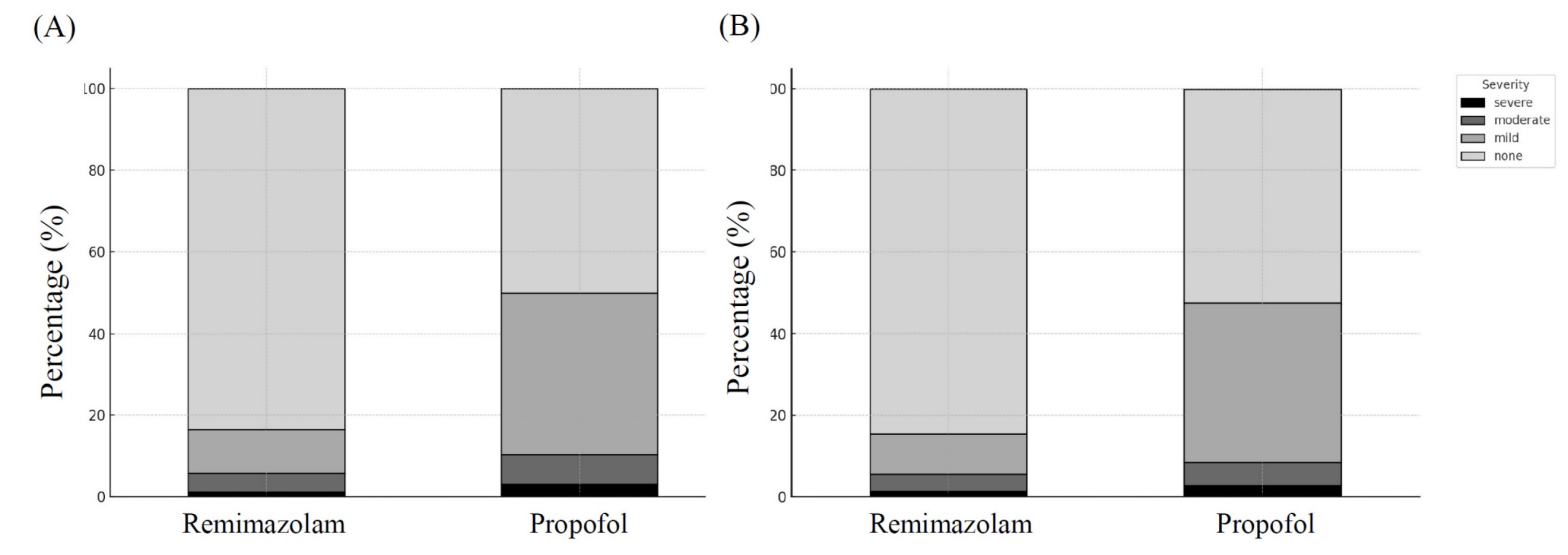
Distribution of intraoperative hypothermia severity between groups before and after propensity score matching. Hypothermia severity was categorized according to the lowest intraoperative core temperature during the first 60 minutes after induction: none (≥ 36.0°C), mild (35.5-35.9°C), moderate (35.0-35.4°C), and severe (< 35.0°C). (A) Distribution before PSM. (B) Distribution after PSM.

The distribution of hypothermia severity also differed significantly between the two groups. After propensity score matching, the proportion of patients without hypothermia remained substantially higher in the remimazolam group than in the propofol group (84.5% vs. 52.1%), whereas moderate-to-severe hypothermia occurred more frequently in the propofol group. The distribution of hypothermia severity is illustrated in Figure 2.

**Figure 3:**
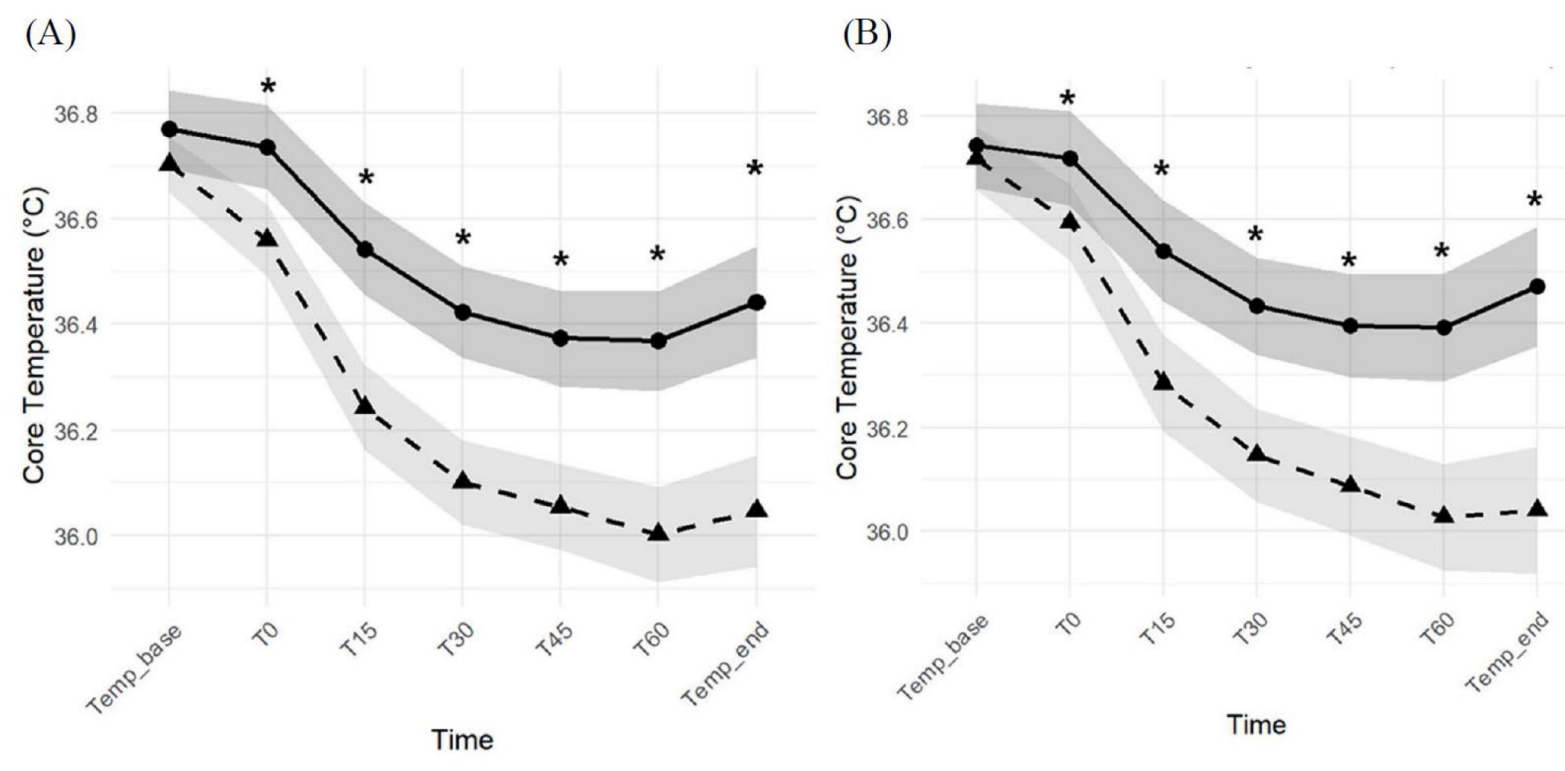
Core temperature trends before (A) and after (B) propensity score matching at each time point (Temp_base = baseline temperature before induction; T0 = immediately after induction; T15, T30, T45, T60 = minutes after induction; Temp_end = end of surgery). Black circles represent the remimazolam group, black triangles represent the propofol group, and shaded areas indicate 95% confidence intervals. Asterisks (*) indicate statistically significant differences between groups at the corresponding time points (P < 0.05).

A repeated measures analysis of variance (RM-ANOVA) showed significant effects of time and group, as well as a significant time-by-group interaction for core temperature (all P < 0.001), both before and after propensity score matching. As shown in Figure 3, the propofol group demonstrated a more pronounced decline in core temperature during the first hour after induction, and post hoc comparisons revealed significantly lower core temperatures than in the remimazolam group at all time points except baseline. These findings indicate that remimazolam was associated with both a smaller decline from baseline and better preservation of absolute core temperature during the early intraoperative period.

Factors associated with intraoperative hypothermia were evaluated using regression analyses (Table 3). In the propensity score-matched cohort, conditional logistic regression stratified by matched pairs showed that the association between propofol use and hypothermia remained robust (adjusted OR 9.00, 95% CI 2.09-38.79; P = 0.003).

Sensitivity analyses using generalized estimating equations with clustering by matched pairs and multivariable adjustment yielded consistent results, confirming propofol use as a strong risk factor for hypothermia (adjusted OR 34.5, 95% CI 5.80-206.0; P < 0.001; **Supplementary Table S1**). In these sensitivity analyses, female sex, higher body mass index, and higher baseline core temperature were associated with a lower risk of intraoperative hypothermia.

## Discussion

In this retrospective propensity-matched cohort study, the use of propofol for induction was associated with a significantly higher incidence and severity of redistribution hypothermia, greater maximum temperature drops, and more frequent PACU warming requirements compared with remimazolam. Importantly, female sex, higher BMI, and higher baseline core temperature were associated with a lower risk of intraoperative hypothermia.

Intraoperative hypothermia, typically defined as a core temperature below 36.0 °C during surgery 18, is a frequent yet often underestimated complication among surgical patients. It has been associated with a wide range of adverse outcomes, including increased risk of cardiovascular events, excessive intraoperative bleeding, higher transfusion requirements, altered drug metabolism, and surgical site infections 2. Hypothermia may also contribute to delayed emergence from anesthesia, prolonged stays in the PACU or intensive care unit (ICU), reduced thermal comfort, and lower patient satisfaction—all of which can lead to increased healthcare costs 19-21. Therefore, exploring anesthetic strategies that mitigate this risk is clinically important.

Propofol, although widely used for induction, produces dose-dependent cardiovascular depression through reduced sympathetic tone, decreased systemic vascular resistance, and venous dilation, leading to reduced preload and cardiac output. This can result in a mean arterial pressure drop of up to 30% after induction 22, 23. Simultaneously, propofol impairs thermoregulatory vasoconstriction, markedly promoting rapid redistribution of core heat to peripheral tissues, as evidenced by a significant reduction in core-to-skin temperature gradient within minutes post-induction 24, 25. This combination of hypotension and vasodilation creates the physiological basis for the redistribution hypothermia frequently observed after propofol induction.

Remimazolam is a recently introduced ultra-short-acting benzodiazepine that has been gaining broader clinical use in both anesthesia and procedural sedation 26. From a thermoregulatory perspective, studies of midazolam—another benzodiazepine—have shown minimal impairment of vasoconstriction thresholds even at high sedative doses, suggesting that remimazolam may likewise help preserve core temperature compared with propofol 15.

Several physiologic considerations should be acknowledged when interpreting the observed core temperature changes. Core temperature measurements alone cannot distinguish among altered central thermoregulatory setpoints, peripheral vasodilation-mediated heat redistribution, and generalized heat loss related to environmental exposure. In this context, the term “redistribution hypothermia” is used to describe the characteristic early decline in core temperature observed within the first hour after anesthetic induction, a phenomenon commonly attributed in the anesthesiology literature to anesthetic-induced peripheral vasodilation and subsequent heat redistribution. However, the relationship between peripheral vasodilation, core-to-peripheral temperature gradients, and net heat loss is physiologically complex, and the mechanistic interpretation of our findings should therefore be made with caution.

Decreased cardiac output or peripheral vasoconstriction may attenuate the magnitude of redistribution hypothermia by reducing heat transfer to the periphery 14. Remimazolam has demonstrated superior cardiovascular stability compared to propofol, although its effects on cardiac output and systemic vascular resistance have varied depending on the doses of remimazolam and propofol applied in the studies 12, 13. In a study in which anesthesia was induced using a remimazolam-to-propofol dose ratio of 1:10, similar to that used in our study, cardiac output did not differ significantly between groups, whereas systemic vascular resistance was better preserved in the remimazolam group 13. Because sevoflurane was used in both groups and titrated to the same PSI target range, inhalational anesthetic-induced vasodilation may have contributed to heat redistribution in both groups; however, it is unlikely to fully explain the observed between-group difference.

In addition, remimazolam has been reported to show a higher vasoconstriction threshold and a faster onset of vasoconstriction than propofol 27. These pharmacologic characteristics may have contributed to attenuation of redistribution hypothermia by more favorably preserving systemic vascular resistance and vascular tone. To date, few studies have directly compared remimazolam and propofol with respect to redistribution hypothermia, a gap our study begins to address.

Our findings suggest that choosing remimazolam instead of propofol for induction of general anesthesia may confer thermoregulatory benefits, particularly in procedures with a high risk of hypothermia, such as urologic surgeries performed in the lithotomy position with large volumes of cold irrigation fluid. The observed reduction in the need for active warming in the PACU with remimazolam could also translate into more efficient postoperative recovery and better utilization of healthcare resources. The observed between-group difference of approximately 0.4 °C at the end of the first hour is also likely to be clinically meaningful. In the perioperative setting, even modest differences in core temperature may determine whether a patient crosses the threshold for hypothermia, particularly in procedures with ongoing heat loss, and may influence postoperative warming requirements. Prewarming should also be considered when interpreting our findings. Previous studies in transurethral surgery have shown that even short periods of active prewarming may attenuate perioperative temperature decline and reduce the incidence of hypothermia. Therefore, the potential thermoregulatory benefit associated with remimazolam should be viewed as complementary to, rather than a substitute for, established preventive strategies such as prewarming.

Another notable finding of our study was the identification of female sex, higher BMI, and higher baseline core temperature as independent protective factors against intraoperative hypothermia. The protective effect of female sex is consistent with previous research. Lopez *et al.* demonstrated that women have higher thermoregulatory thresholds—including vasoconstriction and shivering— by approximately 0.3 °C compared to men, indicating more robust heat-conservation mechanisms in females 28. Similarly, a study of patients undergoing major abdominal surgery identified male sex as a risk factor for intraoperative hypothermia 29, and another investigation in laparoscopic cholecystectomy patients reported a significantly greater mean temperature decrease in male patients because of their higher susceptibility to hypothermia 30. Similarly, a higher BMI may attenuate core temperature decline due to increased insulation from subcutaneous adipose tissue, which reduces heat transfer to the environment 31,32. Baseline core temperature has also been shown to strongly influence intraoperative thermal trajectories, with higher pre-induction temperatures providing a greater margin before reaching the hypothermia threshold 33, 34. Our findings therefore align with existing evidence, reinforcing the relevance of these patient-specific factors in perioperative thermal management strategies.

This study has several strengths. First, the use of propensity score matching minimized confounding by balancing key baseline characteristics between the remimazolam and propofol groups, thereby improving the internal validity of the comparative analysis despite the retrospective design. Secondly, it addresses redistribution hypothermia in urologic surgeries, which inherently carry a high risk of intraoperative heat loss due to lithotomy-position or large-volume cold irrigation. By focusing on this high-risk setting, the findings have practical implications for perioperative thermal management and may contribute to enhanced recovery after surgery (ERAS) strategies, where maintenance of normothermia is known to improve postoperative outcomes. Third, the study population underwent uniform perioperative protocols aside from the induction agent, reducing variability in other factors influencing body temperature. Fourth, continuous and precise measurement of core temperature with a zero-heat-flux thermometry system ensured high reliability of the thermal data.

Nevertheless, certain limitations should be acknowledged. First, the single-center, retrospective design inherently limits generalizability and precludes definitive causal inference. While the study controlled many perioperative variables, unmeasured confounders—such as exact amount of irrigation fluid, degree of surgical exposure, or subtle intraoperative environmental changes—may still have contributed to the observed effects. Although key perioperative proxies such as anesthesia duration, surgical position, administered fluid volume, and blood loss were accounted for, residual confounding related to procedural heterogeneity cannot be completely excluded. Second, although the sample size was adequate to detect differences in the primary outcome, it may not have been sufficient to allow detailed subgroup analyses according to specific urologic procedure type or patient comorbidity profile. Third, direct differentiation of the underlying thermoregulatory mechanisms would have required additional measurements, such as peripheral skin temperature, core-to-peripheral temperature gradients, or vasoconstriction thresholds, which were not obtained in this study. Accordingly, mechanistic interpretations should be made with caution. Fourth, although a standardized perioperative warming protocol was in place at our institution, the actual implementation of warming may have varied among patients. Because of the retrospective design, detailed information regarding the exact timing and duration of active or passive warming, degree of skin exposure and surgical draping, irrigation fluid use, could not be consistently captured. These unmeasured factors may have influenced heat redistribution and core temperature trajectories and should be considered when interpreting the results. Finally, it should be noted that bolus administration of remimazolam for anesthesia induction remains an off-label practice. However, in recent studies 17, 35, 36, a bolus injection of remimazolam has been suggested as an efficient and safe method for anesthesia induction. A 0.2 mg/kg bolus injection of remimazolam for anesthesia induction did not cause significant alterations in hemodynamic parameters or differences in adverse events compared to continuous infusion (6 mg/kg/h and 12 mg/kg/h), while reducing the patient's time to loss of consciousness. Nevertheless, further prospective studies are warranted to establish standardized dosing strategies and to confirm the safety and efficacy of bolus-based induction protocols.

Our findings do not by themselves justify a universal change in standard anesthetic practice. Rather, they suggest that remimazolam may be a reasonable option to consider in patients at increased risk of perioperative hypothermia, particularly those undergoing urologic procedures involving lithotomy positioning, irrigation-related heat loss, or prolonged exposure, as well as in patients in whom even modest hypothermia may be clinically undesirable. These results should be regarded as hypothesis-generating and require confirmation in prospective randomized studies before broader practice recommendations can be made.

In conclusion, in this retrospective propensity-matched cohort, remimazolam was associated with a lower incidence and severity of redistribution hypothermia than propofol in patients undergoing urologic surgery. Prospective randomized controlled trials are warranted to confirm these results, particularly in high-risk surgical populations. Future research should also investigate whether these thermoregulatory benefits lead to improved clinical outcomes, including reduced perioperative complications and enhanced postoperative recovery.

## Supplementary Material

Supplementary table.

## Figures and Tables

**Table 1:** Demographic and perioperative data before and after propensity score matching between remimazolam and propofol group.

	Before propensity score matching					After propensity score matching			
	Remimazolam (n = 85)	Propofol (n = 96)		P value	SMD	Remimazolam (n = 71)	Propofol (n = 71)	P value	SMD
Age (y)	62.0 (11.1)	60.9 (14.0)		0.562	0.087	61.9 (10.3)	61.9 (13.9)	0.978	0.005
Sex, female	23 (27.1)	27 (28.1)		1.000	0.024	20 (28.2)	19 (26.8)	1.000	0.032
Body mass index (kg/m^2^)	24.3 [22.3, 26.7]	23.6 [21.6, 26.1]		0.228	0.202	24.0 [22.2, 26.7]	23.7 [21.8, 26.7]	0.753	0.072
ASA physical status				0.449	0.189			0.940	0.059
I	1 (1.2)	1 (1.0)				1 (1.4)	1 (1.4)		
II	48 (56.5)	63 (65.6)				44 (62.0)	46 (64.8)		
III	36 (42.4)	32 (33.3)				26 (36.6)	24 (33.8)		
Duration of anesthesia (minutes)	100.0 [75.0, 135.0]	100.0 [75.0, 135.0]		0.651	0.118	100.0 [75.0, 132.5]	95.0 [75.0, 130.0]	0.860	0.006
Operation position				0.734	0.075			1.000	0.031
Lithotomy	57 (67.1)	68 (70.5)				50 (70.4)	51 (71.8)		
Supine	28 (32.9)	28 (29.5)				21 (29.6)	20 (28.2)		
Fluid administration (mL)	300.0 [200.0, 400.0]		300.0 [200.0, 450.0]	0.765	0.075	300.0 [200.0, 400.0]	300.0 [200.0, 400.0]	0.943	0.001
Estimated blood loss (mL)	5.0 [2.0, 10.0]		5.0 [3.0, 20.0]	0.054	0.176	5.0 [1.5, 10.0]	5.0 [3.0, 10.0]	< 0.001	0.004
Total dose of induction drug (mg)	15.0 [12.0, 17.0]		120.0 [100.0, 150.0]			15.0 [12.0, 16.0]	120.0 [100.0, 150.0]		
Weight-based dose of induction drug (mg/kg)	0.2 [0.2, 0.2]		2.0 [1.8, 2.0]			0.2 [0.2, 0.2]	1.93 [1.8, 2.0]		
Baseline temperature	36.8 [36.5, 37.0]		36.7 [36.5, 36.9]	0.058	0.219	36.8 [36.5, 37.0]	36.70 [36.5, 37.0]	0.369	0.082
Intraoperative phenylephrine use	27 (31.8)		32 (33.3)	0.948	0.033	55 (77.5)	55 (77.5)	1.000	< 0.001
Intraoperative ephedrine use	68 (80.0)		75 (78.1)	0.900	0.046	23 (32.4)	22 (31.0)	1.000	0.030
									

Values are presented as number (%) or median (interquartile range). ASA, American Society of Anesthesiologists; SMD, standardized mean difference. Baseline temperature; Patient temperature measured in the operating room prior to induction of anesthesia.

**Table 2:** Postoperative outcomes before and after propensity score matching.

	Before propensity score matching			After propensity score matching		
	Remimazolam(n = 85)	Propofol(n = 96)	P value	Remimazolam(n = 71)	Propofol(n = 71)	P value
Incidence of hypothermia	14 (16.5)	48 (50.0)	< 0.001	11 (15.5)	34 (47.9)	< 0.001
Tbaseline - T60	0.4 (0.2-0.6)	0.7 (0.4-0.9)	< 0.001	0.3 (0.2-0.5)	0.6 (0.6-0.9)	0.007
Temperature at 60 minutes after induction	36.4 (36.2-36.6)	36.0 (35.9-36.3)	< 0.001	36.4 (36.2-36.6)	36.0 (35.9-36.3)	0.002
Maximum temperature change	0.5 (0.3-0.7)	0.7 (0.4-0.8)	0.001	0.4 (0.3-0.7)	0.6 (0.4-0.9)	0.011
Warming at PACU	18 (21.2)	51 (52.6)	< 0.001	15 (21.1)	34 (47.9)	0.044

Values are presented as number (%) or median (interquartile range). Tbaseline: Temperature measured in the operating room before induction of anesthesia, T60: Temperature measured 60 minutes after induction of anesthesia, Maximum temperature change: Tbaseline - the lowest temperature during 1 hour after induction of anesthesia. Before propensity score matching, comparisons were performed using the chi-square test or Mann-Whitney U test, as appropriate. After propensity score matching, paired analyses were applied: McNemar's test for binary outcomes and Wilcoxon signed-rank test for continuous

**Table 3:** Logistic regression of hypothermia before and after propensity score matching.

	Before propensity score matching			
	Unadjusted OR (95% CI)	P value	Adjusted OR (95% CI)	P value
Propofol (vs. remimazolam)	5.07 (2.58-10.50)	< 0.001	7.51 (3.16-14.77)	< 0.001
Age (yr)	1.01 (0.98-1.03)	0.630	0.98 (0.95-1.02)	0.284
Sex (Female)	0.59 (0.28-1.19)	0.151	0.46 (0.17-1.18)	0.117
BMI (kg/m^2^)	0.87 (0.79-0.96)	0.008	0.85 (0.74-0.96)	0.013
ASA	0.98 (0.53-1.79)	0.942		
Duration of anesthesia	1.00 (1.00-1.01)	0.172	1.00 (1.00-1.02)	0.240
Supine (vs. lithotomy)	0.86 (0.43-1.67)	0.663		
Estimated blood loss	1.00 (0.99-1.01)	0.828		
Administered fluid	1.00 (1.00-1.00)	0.744		
Use of ephedrine	1.60 (0.74-3.70)	0.249		
Use of phenylephrine	0.54 (0.27-1.07)	0.084	0.61 (0.24-1.51)	0.296
Baseline temperature	0.02 (0.00-0.08)	< 0.001	0.01 (0.00-0.05)	< 0.001
	**After propensity score matching (conditional logistic regression)**			
	**Adjusted OR (95% CI)**		**P value**	
Propofol (vs. remimazolam)	9.00 (2.09-38.79)		0.003	

ASA, American Society of Anesthesiologists; OR, odds ratio; CI, confidence interval. After propensity score matching, conditional logistic regression stratified by matched pairs was used as the primary analysis to account for the paired structure of the data.
