# Supplementary material for: Comparative effects of remimazolam and propofol on intraoperative redistribution hypothermia in urologic surgery: a retrospective propensity-matched cohort study
Source: Int J Med Sci. 2026 Mar 30;23(5):1798–807. doi: 10.7150/ijms.126707 (PMC13133871; doi:10.7150/ijms.126707)
Supplement: Supplementary file 1 — Supplementary table. [file ijmsv23p1798s1.pdf]

Supplementary Table S1 Sensitivity analysis of factors associated with intraoperative hypothermia in the propensity score–matched cohort using generalized estimating equations

Variable	Adjusted OR (95% CI)	P value
Propofol (vs. remimazolam)	34.50 (5.80–206.00)	<0.001
Age (per year)	0.99 (0.95–1.04)	0.790
Sex (female)	0.12 (0.03–0.60)	0.009
BMI (per kg/m²)	0.72 (0.55–0.93)	0.014
ASA class	0.41 (0.07–2.40)	0.320
Duration of anesthesia (per min)	1.00 (0.98–1.03)	0.700
Position (supine vs. lithotomy)	0.67 (0.17–2.58)	0.560
Estimated blood loss (per mL)	1.00 (0.96–1.04)	0.980
Administered fluid (per mL)	1.00 (1.00–1.01)	0.260
Use of ephedrine	5.68 (0.93–34.50)	0.059
Use of phenylephrine	0.41 (0.11–1.49)	0.180
Baseline temperature (per °C)	0.0006 (0.00002–0.014)	<0.001

ASA, American Society of Anesthesiologists; OR, odds ratio; CI, confidence interval

Generalized estimating equations with a logit link and exchangeable correlation structure were used, with clustering by matched pairs. Adjusted odds ratios (ORs) with 95% confidence intervals (CIs) are presented.
